# Macro- and micronutrient disposition in an *ex vivo* model of extracorporeal membrane oxygenation

**DOI:** 10.1186/s40635-014-0029-7

**Published:** 2014-11-22

**Authors:** Kristine Estensen, Kiran Shekar, Elissa Robins, Charles McDonald, Adrian G Barnett, John F Fraser

**Affiliations:** Critical Care Research Group, The Prince Charles Hospital, Rode Road, Brisbane, 4032 Australia; The University of Queensland, Herston Road, Brisbane, 4072 Australia; Institute of Health and Biomedical Innovation, School of Public Health and Social Work, Queensland University of Technology, George Street, Brisbane, QLD 4059 Australia; Adult Intensive Care Services, The Prince Charles Hospital, Rode Rd, Chermside, Brisbane, QLD 4032 Australia

**Keywords:** Extracorporeal membrane oxygenation, Nutrient sequestration, Total parenteral nutrition, Nutritional support

## Abstract

**Background:**

Extracorporeal membrane oxygenation (ECMO) circuits have been shown to sequester circulating blood compounds such as drugs based on their physicochemical properties. This study aimed to describe the disposition of macro- and micronutrients in simulated ECMO circuits.

**Methods:**

Following baseline sampling, known quantities of macro- and micronutrients were injected post oxygenator into *ex vivo* ECMO circuits primed with the fresh human whole blood and maintained under standard physiologic conditions. Serial blood samples were then obtained at 1, 30 and 60 min and at 6, 12 and 24 h after the addition of nutrients, to measure the concentrations of study compounds using validated assays.

**Results:**

Twenty-one samples were tested for thirty-one nutrient compounds. There were significant reductions (*p* < 0.05) in circuit concentrations of some amino acids [alanine (10%), arginine (95%), cysteine (14%), glutamine (25%) and isoleucine (7%)], vitamins [A (42%) and E (6%)] and glucose (42%) over 24 h. Significant increases in circuit concentrations (*p* < 0.05) were observed over time for many amino acids, zinc and vitamin C. There were no significant reductions in total proteins, triglycerides, total cholesterol, selenium, copper, manganese and vitamin D concentrations within the ECMO circuit over a 24-h period. No clear correlation could be established between physicochemical properties and circuit behaviour of tested nutrients.

**Conclusions:**

Significant alterations in macro- and micronutrient concentrations were observed in this single-dose *ex vivo* circuit study. Most significantly, there is potential for circuit loss of essential amino acid isoleucine and lipid soluble vitamins (A and E) in the ECMO circuit, and the mechanisms for this need further exploration. While the reductions in glucose concentrations and an increase in other macro- and micronutrient concentrations probably reflect cellular metabolism and breakdown, the decrement in arginine and glutamine concentrations may be attributed to their enzymatic conversion to ornithine and glutamate, respectively. While the results are generally reassuring from a macronutrient perspective, prospective studies in clinical subjects are indicated to further evaluate the influence of ECMO circuit on micronutrient concentrations and clinical outcomes.

## Background

Extracorporeal membrane oxygenation (ECMO) is increasingly being utilized as a viable supportive treatment modality for acute severe refractory cardiorespiratory failure [1-3]. It can be used short term where recovery of the lungs or heart is anticipated, or as a bridge to long-term mechanical cardiac support or lung transplantation. Patients supported on ECMO are not only critically unwell but are often at cross roads while they await organ recovery or further definitive therapy [1]. Despite controversies [4] around what constitutes optimal nutritional support in an intensive care unit (ICU), preserving nutritional status and overall physical condition is an important aspect of their management [5].

There is growing evidence that malnutrition in the critically ill influences morbidity and mortality [6-8]. Patients may receive ECMO support for weeks or may have had chronic disease which impacts their nutritional status pre-morbidly. Available data indicates that feed intolerance may occur in up to 38% of the ECMO patients [5] and that patients may be receiving as little as 50% of their nutritional requirements due to enteral feed intolerance [9] resulting in large caloric deficit. In this setting, sequestration if any of vital nutrients in ECMO circuits may lead to further nutritional debilitation.

The ECMO circuit is not, however, a passive conduit for the blood and may sequester a variety of circulating compounds such as drugs [10-12] and possibly nutrients, effectively reducing the bioavailability of these compounds. Other *ex vivo* studies in cardiopulmonary bypass circuits have demonstrated that these circuits can sequester trace elements [13]. Hence, it is therefore plausible that there may be clinically relevant interactions between the ECMO circuit and macro-/micronutrients. However, to date, there is no data pertaining to nutrient disposition in the ECMO circuit.

The aim of this 24-h single-dose study was to identify any significant reduction in circulating concentrations of macronutrients (glucose, amino acids and fatty acids) or micronutrients (fat soluble vitamins, water soluble vitamins, selenium, copper, zinc and manganese) in an *ex vivo* ECMO circuit model primed with the fresh human whole blood.

## Methods

Ethics approval was obtained from the local Human Research Ethics Committee (HREC/12/QPCH/90).

### ECMO circuits

The methods for the development of the *ex vivo* model of ECMO used in the experiment have been published previously [14]. Briefly, three new ECMO circuits were used (Maquet Cardiopulmonary AG, Hechinger Strabe, Germany). Each circuit had an identical composition: Bioline tubing, a PLS Quadrox D oxygenator and a Rotaflow pump head. A reservoir bladder (R-38; Medtronic Pty Ltd., Minneapolis, MN, USA) was added to contribute compliance to the system and thus, allow blood sampling from a closed circuit. The three circuits were initially primed with 0.9% saline (Baxter Healthcare, Toongabbie, NSW, Australia). Five thousand international units of Porcine mucous heparin (Pfizer Pty Ltd., Perth, Australia) was added to the circuits. The fresh human whole blood (6 days old) was then added to the circuit in exchange for the saline prime. Circuits were volume pressurized to obtain post-oxygenator pressures of 230 to 250 mmHg.

The final volume of the pressurized circuit was 819 ± 82 mL with a mean haemoglobin value of 70 ± 2 g/L. Oxygen tension in the circuit blood was maintained between 150 to 200 mmHg (mean 175 ± 8 mmHg). Circuit temperature was maintained at 37°C. The pH of the circulating blood was maintained in the range of 7.25 to 7.55 by use of carbon dioxide gas or sodium bicarbonate solution added to the circuit. The pH, pO_2_, pCO_2_, potassium and lactate were checked at baseline, 6 h and at the conclusion of the experiment (24 h) to ensure consistency between all three circuits. Flow rates in each circuit were maintained at 4 to 5 L/min. Ambient temperature and light conditions were identical for all three circuits during the experiment.

### Preparation and injection of nutrient solutions

Standard parenteral nutrition (PN) solution was prepared from a ready-to-mix triple phase bag containing amino acids, glucose, lipids and electrolytes (4QH.01TC, Baxter Healthcare, Toongabbie, NSW, Australia) as per manufacturer's recommendations. One dose each of trace element solution (10 mL Baxter MTEFE) and vitamin powder (Baxter Cernevit reconstituted in 5 mL water) was added to the PN bag through a sterile port. Each PN bag was agitated for 3 min to ensure thorough distribution of contents while being covered by a light impermeable bag provided by the manufacturer. After reconstitution, 10 mL of this solution was extracted in a clean 10-mL syringe and then injected into the *ex vivo* circuit at the post-oxygenator site. This dose was selected to emulate typical concentrations observed in an ECMO patient receiving total PN (70 mL/h of 3:1 TPN premix solution) over a 24-h period. This was necessary to exclude any concentration-dependent sequestration.

### Blood sample collection and nutrient assays

**Figure 1:**
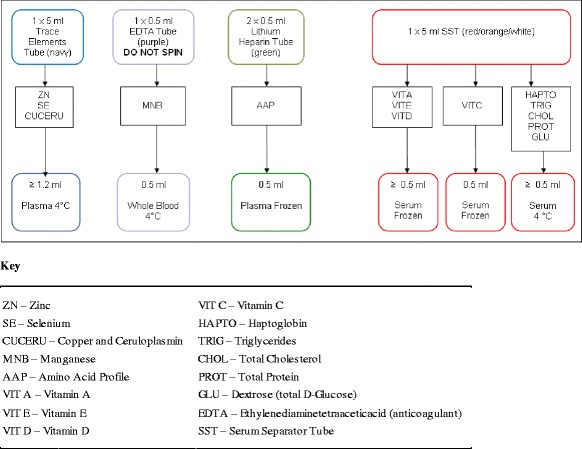
**Sample collection schedule.**

A 3-min period of equilibration was allowed for each circuit at a flow rate of 4 L/min prior to injection of PN solution. Blood samples (10 mL each) were collected from each circuit from the post-oxygenator site at time-points: baseline (after equilibration), 1 min (after addition of PN), 30 and 60 min and 6, 12 and 24 h. Figure 1 details the collection of samples using light protective precautions to prevent photodegradation. Samples were analysed using nutrient assays as outlined in Table 1.

**Table 1:** **Nutrient assay methods**

**Analyte**	**Methodology**
Zinc (serum)	The Wako Zinc (Zn) test WAKO Germany
Selenium	Atomic absorption spectrometry
	Varian Model 280Z Zeeman GFAAS
Manganese (whole blood Mn)	Atomic absorption spectrometry
	Varian Model 280Z Zeeman GFAAS
Copper	Atomic absorption spectrometry
	Varian Model 280Z Zeeman GFAAS
Vitamin E (α-tocopherol)	Reversed-phase HPLC with multiwavelength detection
	Waters™ 2690/5 HPLC Alliance Separations System
Vitamin A (retinol and retinol esters)	Reversed-phase HPLC with multiwavelength detection
	Waters™ 2690/5 HPLC Alliance Separations System
Plasma amino acids	Reverse phase UPLC and TUV detector
	MassTrak™ Amino Acid Analysis Kit
	Waters™ Acquity Ultra Performance LC System
Vitamin C (ascorbic acid)	Spectrophotometric endpoint assay
	Horiba ABX Diagnostics Pentra 400
Vitamin D (25-OH vitamin D2 and D3)	Liquid chromatography-tandem mass spectrometry
	TECAN EVO2. Waters™ Acquity Ultra Performance LC system
Haptoglobin	Turbidimetric method
	Beckman Coulter Synchron Clinical System
Triglycerides	Timed-endpoint method
	Beckman Coulter Synchron Clinical System
Cholesterol	Timed-endpoint method
	Beckman Coulter Synchron Clinical System
Protein (total protein)	Timed-endpoint biuret method
	Beckman Coulter Synchron Clinical System
Glucose (serum glucose)	Glucose oxidase method
	Beckman Coulter Synchron Clinical System

### Statistical analysis

We used a mixed model with a random intercept for each circuit to control for the nonindependence of results from the same circuit. The nutrient concentrations in samples drawn at 1-min interval following the injection of PN solution into the circuit were included in the model as a baseline. The key predictor variable was time in hours which we assumed to be linear. All analyses were made using R version 3.0.2. This model accounts for the repeated responses from the same circuit using a random intercept. The concentration of nutrient versus time curves (mean ± SEM) were plotted using GraphPad Prism Version 5.03.

## Results

There were no circuit complications. There were no significant differences between oxygen or carbon dioxide tensions, pH, temperature, electrolyte composition and degree of hemolysis between circuits. Circuit parameters are described in Table 2.

**Table 2:** **Circuit parameters**

**Circuit**	**1**	**2**	**3**
Total circuit volume (mL)	857	738	861
Crystalloid volume (mL)	325	203	374
Whole blood volume (mL)	532	535	487
Age of whole blood (days)	6	6	5
Temperature (C)	36.5	36.7	36.6
Heparin dose (IU)	5,000	5,000	5,000
Calcium chloride dose (mL)	10	10	10
Calcium dose (mmol)	3.4	3.4	3.4
Oxygen (L/min)	6	6	6

**Figure 2:**
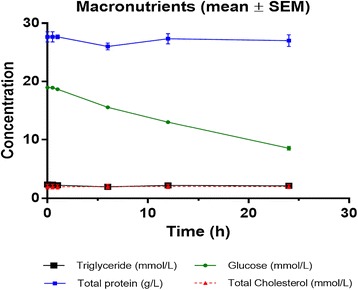
**Glucose, lipid and total protein concentrations in the** ***ex vivo*** **ECMO circuit over the 24-h study period.**

**Figure 3:**
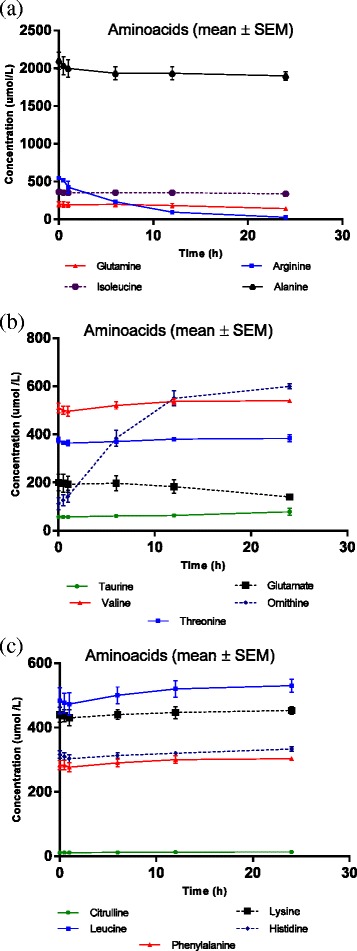
**Amino acid concentrations in the** ***ex vivo*** **ECMO circuit over the 24-h study period (a-c).**

**Figure 4:**
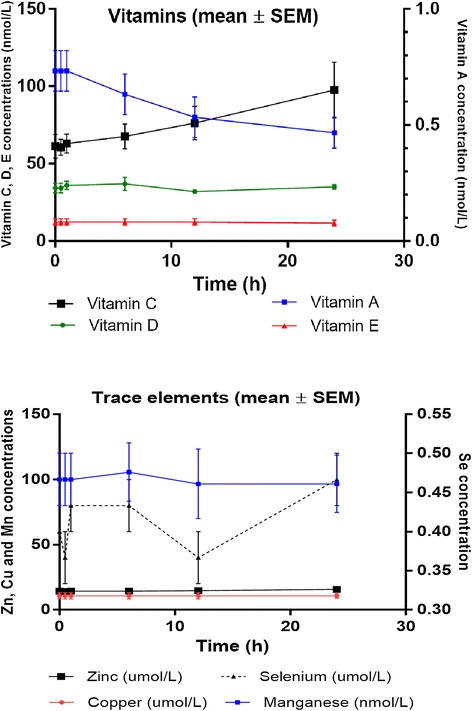
**Circulating trace element and vitamin concentrations in the** ***ex vivo*** **ECMO circuit over the 24-h study period.**

Twenty-one samples were analysed for thirty-one nutrient compounds. Linear changes in hourly nutrient concentrations in the ECMO circuit are presented in Table 3. The 95% CIs for mean hourly changes for a majority of nutrients indicate minimal variability in nutrient concentrations between circuits. Concentration versus time curves for the test compounds are graphed in Figures 2,3,4. There were significant reductions (*p* < 0.05) in circuit concentrations of some amino acids [alanine (10%), arginine (95%), cysteine (14%), glutamine (25%) and isoleucine (7%)], vitamins [A (42%) and E (6%)] and glucose (42%) over 24 h. Significant increases in circuit concentrations (*p* < 0.05) were observed over time for most amino acids, zinc and vitamin C. There were no statistically significant reductions in total proteins, triglycerides, total cholesterol, selenium, copper, manganese and vitamin D concentrations within the ECMO circuit over a 24-h period. While the reductions in glucose concentrations and an increase in other macro- and micronutrient concentrations probably reflect cellular metabolism and breakdown, the decrement in arginine and glutamine concentrations may be attributed to their enzymatic conversion to ornithine and glutamate, respectively. There was no significant correlation between physicochemical properties such as molecular weight, polarity and solubility on circuit disposition.

**Table 3:** **Linear changes over time in nutrient levels**

**Nutrient**	**Units**	**Mean**	**Lower**	**Upper**	***p*** **value**
Macronutrients					
Glucose*	mmol/L	−0.44	−0.47	−0.41	*<*0.001
Total protein	g/L	−0.02	−0.08	0.04	0.54
Total cholesterol	mmol/L	0.00	−0.00	0.00	0.60
Triglycerides	mmol/L	−0.00	−0.02	0.00	0.45
Alanine*	μmol/L	−4.87	−7.78	−1.95	0.008
Arginine*	μmol/L	−19.6	−25.4	−13.83	*<*0.001
Citrulline	μmol/L	0.10	0.04	0.15	*<*0.003
Cystine*	μmol/L	−0.07	−0.16	0.01	0.12
Glutamate	μmol/L	3.86	3.23	4.48	*<*0.001
Glutamine*	μmol/L	−2.34	−3.29	−1.38	*<*0.001
Glycine	μmol/L	−0.04	−3.23	3.14	0.97
Histidine	μmol/L	1.14	0.78	1.51	*<*0.001
Isoleucine*	μmol/L	−0.61	−1.08	−0.13	0.02
Leucine	μmol/L	2.43	1.69	3.17	*<*0.001
Lysine	μmol/L	0.8	0.34	1.410	0.006
Ornithine	μmol/L	20.64	15.42	25.85	*<*0.001
Phenylalanine	μmol/L	1.04	0.57	1.51	0.001
Proline	μmol/L	0.24	−0.60	1.08	0.57
Serine	μmol/L	0.32	−0.20	0.83	0.23
Taurine	μmol/L	0.90	0.43	1.37	0.002
Threonine	μmol/L	0.83	0.35	1.31	0.007
Tyrosine	μmol/L	0.62	0.46	0.77	*<*0.001
Valine	μmol/L	1.86	1.01	2.71	*<*0.001
Trace elements					
Copper	μmol/L	−0.00	−0.02	0.02	1
Manganese	nmol/L	−0.20	−0.51	0.1	0.23
Selenium	μmol/L	0.00	−0.00	0.01	0.21
Zinc	μmol/L	0.06	0.04	0.09	*<*0.001
Vitamins					
Vit A*	nmol/L	−0.01	−0.01	−0.01	*<*0.001
Vit C	nmol/L	1.53	1.01	2.06	*<*0.001
Vit D	nmol/L	−0.05	−0.22	0.13	0.62
Vit E*	nmol/L	−0.03	−0.04	−0.01	0.003

Using a mixed model with post-parenteral nutrition solution injection plasma nutrient concentrations as the baseline and a random intercept for each circuit. Mean changes and 95% confidence intervals. The estimates in the table are per hour. For example, for alanine, the mean loss in 4.87 units per hour with a 95% confidence interval of −7*.*78 to −1.95. Nutrients with significant reductions in concentrations over 24 h are marked in asterisk (*).

## Discussion

To our knowledge, this study represents the first investigation of macro- and micronutrient disposition in *ex vivo* ECMO circuits. Significant alterations in macro- and micronutrient concentrations were observed in this study. Most significantly, there is potential for circuit loss of essential amino acid isoleucine and lipid soluble vitamins (A and E) in the ECMO circuit, and the mechanisms for this need further exploration. While the results are generally reassuring from a macronutrient perspective, prospective studies in clinical subjects are indicated to further evaluate the influence of ECMO circuit on micronutrient concentrations and clinical outcomes.

The findings of this preliminary study that identifies nutrients that risk being lost in ECMO circuits are significant for the following reasons. In the absence of circuit sequestration of most macro- and micronutrients, it can be assumed that the nutrient bioavailability from enteral feeds should be almost entirely related to feed tolerance and absorption and clinicians can focus on improving either of these as feasible. Equally, when feed intolerance is intractable, PN in standard doses may be a viable option. Most importantly, identifying the macro- and micronutrients that are most affected by the addition of an extracorporeal circuit to a critically ill patient may allow better nutritional surveillance and/or supplementation as appropriate. While the tested nutrients play an important role in health and disease, it is unlikely that the adverse clinical outcomes if any of circuit nutrient loss and benefits if any of supplementing at risk nutrients will ever be tested in a randomised trial design and is probably not of sufficient priority. However, minimising the biological burden from mechanical assist device therapy has the potential to improve patient outcomes, and this study is one step in that direction.

Nutrient compounds possess unique physicochemical properties - size, charge and hydrophobicity vary significantly within and between each nutrient group [15]. Glucose is polar, ionic and hydrophilic; the amphipathic fatty acids possess both hydrophilic and lipophilic properties; and amino acids can be measured on a hydrophobicity index due to the varying solubilities of different amino acids in water and in varying pH conditions [15-17]. The majority of vitamins are hydrophilic with the exception of vitamins A, D, E and K, which are lipophilic; trace elements such as copper, manganese and zinc are positively charged cations; and selenium is a negatively charged anion [15]. No clear correlation could be established between physicochemical properties such as polarity, solubility or molecular weight and circuit behaviour of nutrients.

In this setting, interpretation of the alterations in individual nutrient concentrations is challenging as several physical, chemical, environmental and other unknown factors may be at play. The decrement in arginine and glutamine concentrations may be attributed to their enzymatic conversion to ornithine and glutamate, respectively [18-21]. Similarly, the decrease in glucose concentrations over time may be explained in part by cellular metabolism. The reductions in concentrations of amino acids such as cysteine, isoleucine and alanine and vitamins (A and E) may be explained by instability, degradation, circuit sequestration or oxidative mechanisms [22]. It is possible that lipophilicity of vitamins A and E may affect their circuit disposition; however, this was not a consistent trend as another lipophilic vitamin D was relatively unaffected. We also hypothesise that an increase in concentrations of many other nutrients in a closed circuit probably represents breakdown of cellular and noncellular components of the whole blood and detailed evaluation of mechanisms responsible for alterations in individual nutrient concentrations is beyond the scope of this experiment.

Regardless of the cause, the net result is that some nutrients are significantly lost in the ECMO circuit and the clinical relevance of this remains unclear. While saturation of circuit binding sites over time may partly alleviate circuit loss of nutrients, the circuit loss from other mechanisms such as chemical and physical degradations, oxidation etc. may continue over the duration of ECMO, as up to second/third of patient's cardiac output may be exteriorised every minute for gas exchange and mechanical circulatory support [23]. Even though enteral or parenteral nutritional support and endogenous synthetic/metabolic mechanisms ensure an ongoing supply of macro- and micronutrients into the blood stream during ECMO, its unclear how much of their bioavailability is affected by ongoing losses in the circuit. In addition, the circuit loss of some nutrients can further be compounded by critical illness factors [24-26] such as inflammation, multiple organ dysfunction, fluid shifts and capillary leaks that may further affect their bioavailability. Interestingly, this may be the fate of many circulating endogenous compounds such as hormones and many other biologically active substances which are neither monitored nor replaced during ECMO. This calls for further clinical studies to further elucidate the biological impact of the ECMO circuit on circulating nutrients.

This study has limitations. We attempted to replicate the clinical scenario *ex vivo*, which allowed us to specifically study the interactions between the nutrients and the ECMO circuit under physiologic conditions in the absence of patient and pathological factors. This single-dose study, however, contrasts with the clinical scenario wherein there is ongoing delivery of nutrients into the blood stream and ECMO support may be continued for days to weeks. A continuous infusion of PN solution into the fully primed, noncompliant circuit was not feasible. Although a reservoir bladder which is seldom used in adult patients may have added compliance to the circuit, it could have further confounded our results by sequestering test compounds or creating areas of stasis, both of which are undesirable. The absence of circulating drugs, other blood components and metabolites in our experiment may also influence nutrient interactions with the components of the circuit. We did not test nutrient losses in physiologic controls (fresh human whole spiked with equivalent dose of PN solution, stored at 37°C over 24 h) to measure nutrient losses if any over time, independent of the ECMO circuit which to an extent limited the interpretation of our results.

However, this study delineates the circuit behaviour of thirty-one vital nutrients and serves as a necessary first step in identifying at risk nutrients and identifies areas for further research.

## Conclusions

Except for essential amino acid isoleucine and vitamins (A and E), most other macro- and micronutrients tested in this study were stable in the ECMO circuit over 24 h. Exteriorisation of large amounts of patient blood volume for ECMO is life-saving but may lead to circuit loss of vital nutrients. Critical illness may further exacerbate these circuit interactions of nutrients, and future studies should further evaluate altered nutrient disposition during ECMO and their potential impact on clinical outcomes.

## Abbreviations

ECMO: extracorporeal membrane oxygenation
EN: enteral nutrition
MTEFE: micronutrient and trace elements with iron
PN: parenteral nutrition
SEM: standard error of the mean
TPN: total parenteral nutrition
